# An older patient with active ulcerative colitis and coronavirus disease 2019 (COVID-19) pneumonia successfully treated with the combination of anti-TNFα therapy and azathioprine

**DOI:** 10.1007/s12328-022-01737-y

**Published:** 2022-11-23

**Authors:** Tsukasa Yamakawa, Keisuke Ishigami, Sae Ohwada, Tomoe Kazama, Daisuke Hirayama, Shinji Yoshii, Hiro-o Yamano, Hiroshi Nakase

**Affiliations:** 1 Department of Gastroenterology and Hepatology, Sapporo Medical University School of Medicine, S-1 W-16, Chuo-Ku, Sapporo, 060-8543 Japan; 2 Department of General Medicine, Sapporo Medical University School of Medicine, Sapporo, Japan

**Keywords:** Ulcerative colitis, COVID-19, Anti-TNFα antibody

## Abstract

A 77-year-old patient with ulcerative colitis (UC) was transferred to our department because of worsening bloody diarrhea and abdominal pain, which was consistent with a UC flare. Two days after admission, she complained of cough and high fever. The polymerase chain reaction (PCR) test for severe acute respiratory syndrome coronavirus 2 (SARS-CoV-2) was positive, and a computed tomography showed pneumonia in the left lobe, consistent with coronavirus disease 2019 (COVID-19) pneumonia. However, frequent bloody diarrhea and abdominal pain due to the UC flare persisted; therefore, an additional immunosuppressive agent needed to be considered. We initiated infliximab biosimilar (IFX-BS), and her abdominal symptoms improved. However, they deteriorated after the second IFX-BS infusion. After confirming that the patient was negative for SARS-CoV-2 by PCR, we administered a combination of azathioprine and IFX-BS. The combination treatment improved her intestinal symptoms without worsening COVID-19 pneumonia. She has remained in remission for over a year since her discharge.

## Introduction

Since December 2019, the novel coronavirus disease 2019 (COVID-19) caused by severe acute respiratory syndrome coronavirus 2 (SARS-CoV-2) has caused a worldwide pandemic [1, 2]. COVID-19 has various clinical presentations, with some patients being asymptomatic or having mild symptoms. However, the disease can become severe and result in hospitalization, respiratory failure, or death, with fatality rates ranging from 2.3 to 7.2% [3–5].

Inflammatory bowel disease (IBD), including Crohn’s disease and ulcerative colitis (UC), is a general term for chronic or relapsing inflammatory diseases of the gastrointestinal tract [6]. Many IBD patients require treatment with immunosuppressive drugs, which can increase the risk of infection [7]. There is great concern for patients with IBD to develop severe pneumonia owing to immunosuppressive states [5]. However, there are few reports on the initiation of immunosuppressive drugs in patients with IBD in the midst of COVID-19 infection. Herein, we present an older patient with active UC and COVID-19 pneumonia who was successfully treated with an anti-tumor necrosis factor (TNFα) agent and azathioprine (AZA) while appropriately monitoring the COVID-19 infection.

## Case report

**Fig. 1:**
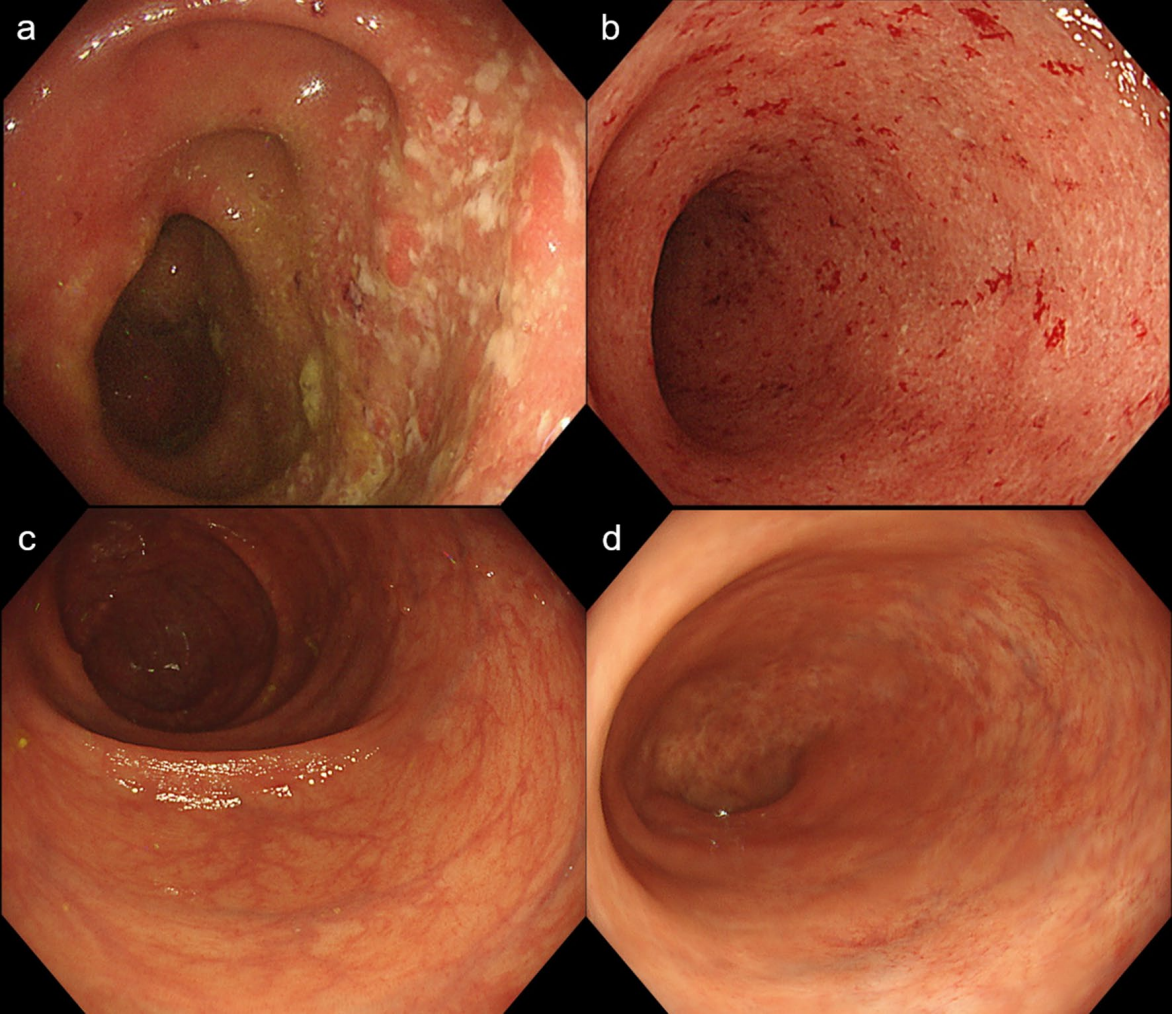
Colonoscopy revealing continuous loss of vascular marking, erosions, and bleeding in the entire colon. **a** the cecum and **b** the rectum Mucosal healing with scarring was confirmed after combination treatment with an anti-TNFα agent and azathioprine **c** the cecum and **d** the rectum

A 77-year-old woman was diagnosed with UC 6 months previously. After she was started on oral 5-aminosalicylic acid (5-ASA), her abdominal symptoms improved. However, her UC flared, and she was transferred to our department for further treatment. Blood tests showed a hemoglobin level of 10.9 g/dL, a C-reactive protein level of 1.89 mg/dL, and an erythrocyte sedimentation rate of 44 mm/h (Table 1). Despite the increased serum level of PR3-anti-neutrophil cytoplasmic antibody (ANCA) in this case, the patient showed no renal dysfunction, abnormal urinalysis, cutaneous symptoms, and neurologic symptoms related to ANCA-associated vasculitis. Stool culture and tests for pathogens, including *Clostridioides difficile*, were all negative. Although the inflammation was limited to the rectum at onset, it extended into the entire colon with a Mayo endoscopic subscore of 2 (Fig. 1a, b). Histological examination of biopsies in each part of the colon confirmed the destruction of the glandular epithelium with crypt abscess and severe inflammatory cell infiltration. These clinical and endoscopic findings were consistent with those of moderate UC.

**Table 1:** Laboratory data on admission

CBC		Biochemistry		Serological test	
WBC	6,800/μl	TP	6.5 g/dl	IgG	2336 mg/dL
Neut	64.0%	Alb	2.2 g/dl	IgM	53 mg/dL
Lym	20.0%	T-bil	0.4 U/L	IgA	412 mg/dL
RBC	3.42 × 10^6^/μl	AST	20 U/L	PR3-ANCA	86.1 U/ml
Hb	10.9 g/dl	ALT	16 U/L	MPO-ANCA	(−)
Hct	32.8%	LDH(IFCC)	232 U/L		
Plt	39.3 × 10^4^/μl	ALP(JSCC)	136 U/L		
		γ-GTP	22 U/L		
Infectious marker		AMY	34 U/L	Coagulation	
HBs-Ag	(−)	ChE	88 U/L	PT	11.6 s
HCV-Ab	(−)	BUN	6 mg/dL	PT(%)	101.3%
RPR	(−)	Cr	0.57 mg/dL	APTT	31.3 s
TPLA	(−)	FBS	86 mg/dL	Fibrinogen	464 mg/dL
CMV-IgM	(−)	HbA1c(NGSP)	6.5%	D-dimer	2.6 μg/mL
CMV-IgG	(+)	CRP	1.89 mg/dL		
CMV-Antigenemia	(−)	Ferritin	423.0 ng/mL	ESR	44 mm/hr
Tb (ELISpot)	(−)	SAA	323.8 μg/mL		
SARS-CoV-2 PCR	(−)

**Fig. 2:**
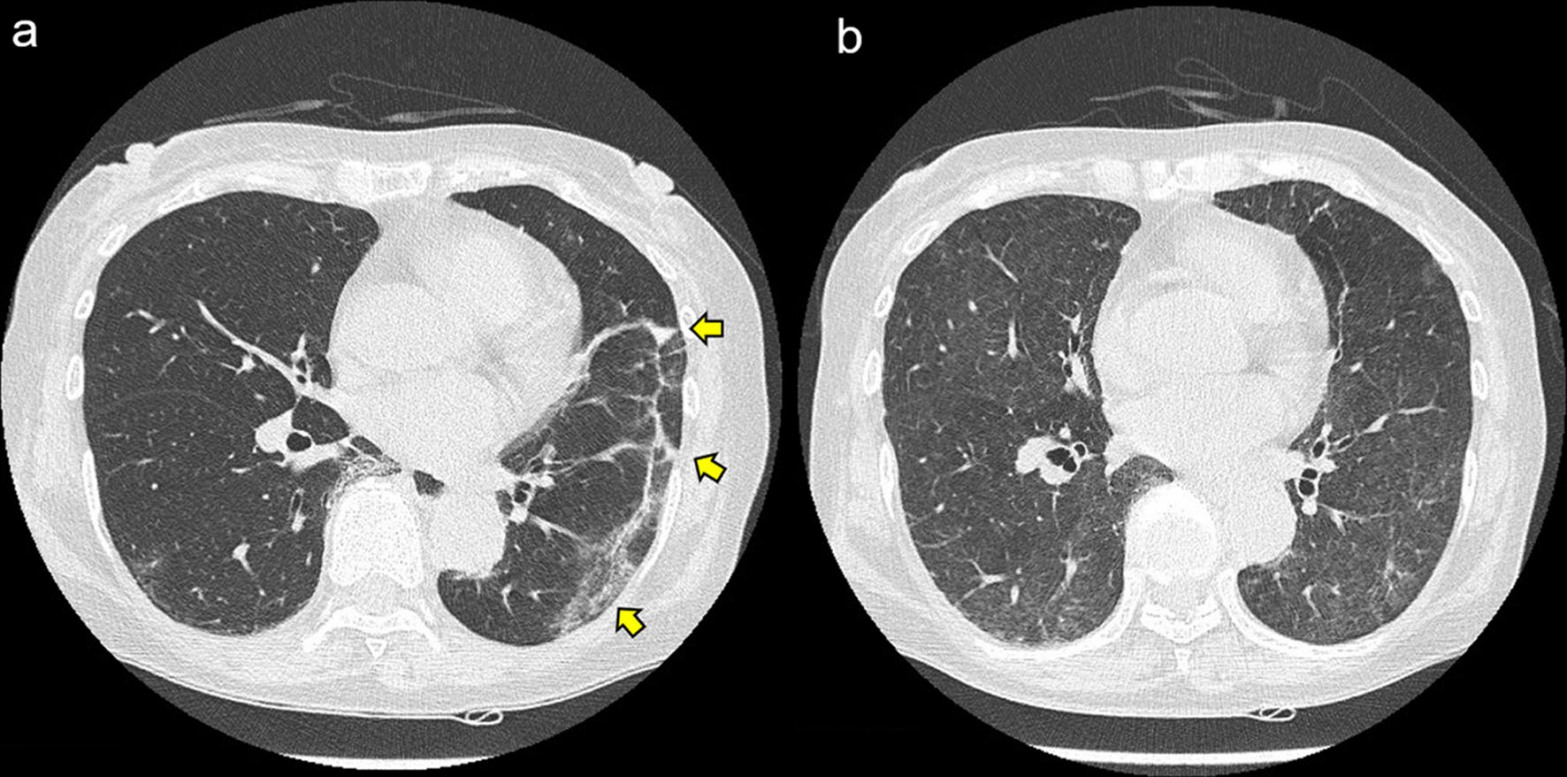
Chest computed tomography scan showing a ground-glass appearance with consolidation (yellow arrows) in the subpleural areas of the left lobe at the onset of COVID-19 (**a**), and the findings have disappeared in a month (**b**)

**Fig. 3:**
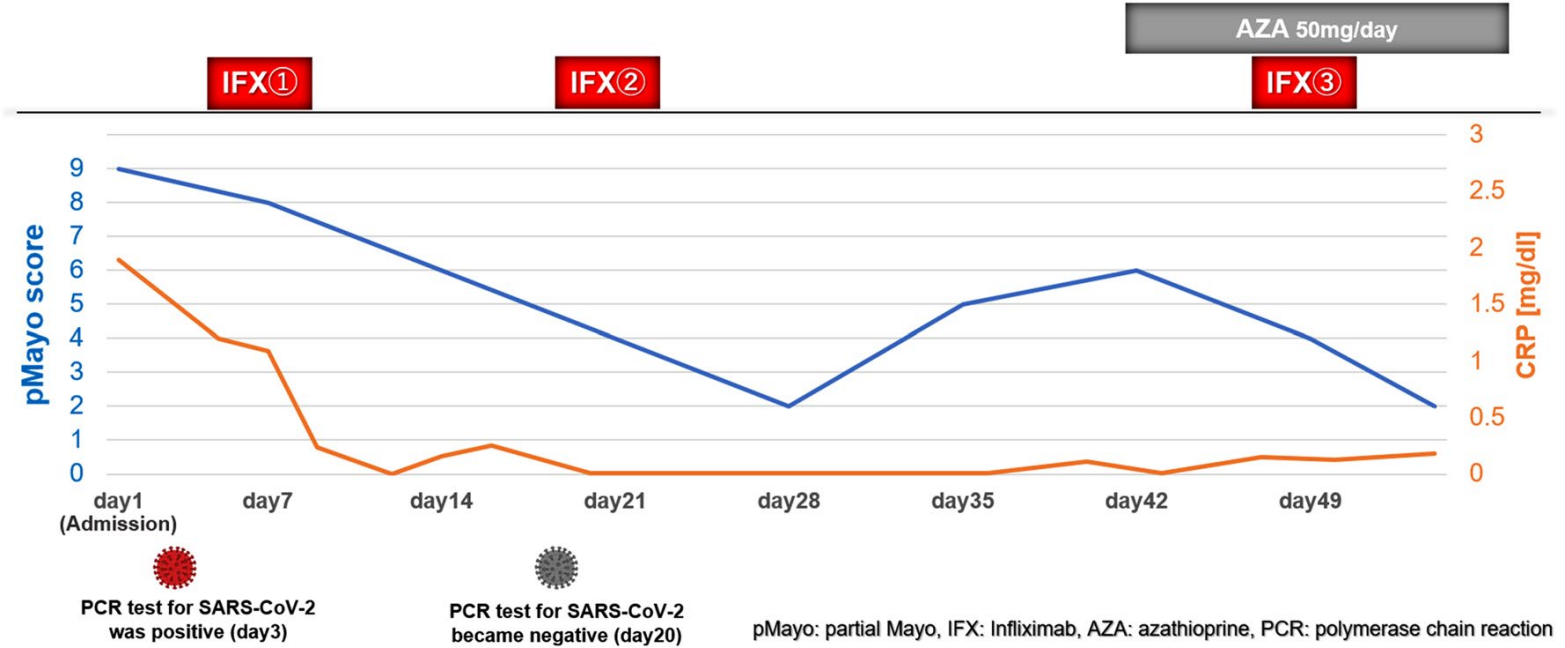
Clinical course of this patient

Two days after admission, the patient complained of a cough and fever of 38.5 °C with a decline in peripheral capillary oxygen saturation (SpO2) to 93%. Chest radiography showed a slight reticular pattern, and computed tomography showed a ground-glass appearance with consolidation in the subpleural areas of the left lobe (Fig. 2). The polymerase chain reaction (PCR) test for SARS-CoV-2 was positive, and we diagnosed her with COVID-19 pneumonia. The patient did not undergo vaccination against SARS-CoV-2 prior to the COVID-19 infection. According to the WHO COVID-19 disease severity categorization, the patient was classified as a moderate case. Although the patient had COVID-19 pneumonia, frequent bloody diarrhea and abdominal pain due to the UC flare persisted; therefore, additional immunosuppressive agents needed to be considered. We initiated infliximab biosimilar (IFX-BS) (5 mg/kg), which improved the clinical activity of UC from a partial Mayo score of 9 to 2. However, her intestinal symptoms gradually deteriorated after the administration of the second dose of IFX-BS. We confirmed that the patient was negative for SARS-CoV-2 by PCR and started 50 mg/day of AZA before the next IFX-BS infusion. After receiving a combination of IFX-BS and AZA, her intestinal symptoms improved (Fig. 3). Regarding her COVID-19, she temporarily required oxygen administration; however, her respiratory symptoms improved without additional therapeutic agents for COVID-19 pneumonia. Follow-up endoscopy performed 1 year and 3 months after the first IFX-BS administration showed mucosal healing with scarring (Fig. 1c, d), and she has been in remission for over a year.

## Discussion

Here, we present a case of an older patient with moderately active UC and COVID-19 pneumonia, who was successfully treated with an anti-TNFα agent and AZA, while appropriately monitoring the COVID-19 infection.

COVID-19, caused by SARS-CoV-2, is an infectious disease that affects the respiratory system. There is convincing evidence that the rates of both morbidity and mortality related to COVID-19 are higher in older patients with comorbidities [7]. A multicenter registry cohort study, Japan COVID-19 surveillance in IBD (J-COSMOS), also revealed that older age, higher body mass index, and steroid use were independent risk factors for COVID-19 severity [8]. The mortality rate was 8.3% in IBD patients over 70 years of age and 20.2% in those over 80 years of age [3, 5].

Patients with IBD are treated with 5-ASA, corticosteroids, thiopurines, and molecular-targeting agents, depending on the extent and severity of the disease [9]. Therefore, both physicians and patients with IBD are concerned about developing COVID-19 in IBD patients while on immunomodulatory therapy. Based on the Surveillance Epidemiology of Coronavirus Under Research Exclusion for Inflammatory Bowel Disease (SECURE-IBD) and J-COSMOS data, older age and the use of steroids contributed to the severity of COVID-19, while anti-TNFα agents did not [3, 5, 8]. Moreover, anti-TNFα agents have been reported to control the multisystem inflammatory syndrome related to COVID-19 [10]. Therefore, we first selected anti-TNFα therapy for this patient. Although patients with COVID-19 primarily experience respiratory symptoms, several reports have indicated how the severity of COVID-19 and gastrointestinal symptoms are associated [11, 12]. The angiotensin-converting enzyme 2 receptor to which SARS-CoV-2 binds is highly expressed in the small and large intestines, suggesting that SARS-CoV-2 may directly affect the intestinal epithelium and cause gastrointestinal symptoms [13, 14]. Furthermore, SARS-CoV-2 infection induces several proinflammatory cytokines such as TNFα, interleukin (IL) -1, and IL-6, and activates the NLR family pyrin domain containing 3 (NLRP3) inflammasome pathway which is important for the host’s defense during viral infection [15–17]. These data suggest that SARS-CoV-2 infection may have contributed to the UC flares in the present case.

Another important point to note in this case is that the administration of an anti-TNFα agent did not worsen her COVID-19 pneumonia. Regarding the relationship between TNFα and COVID-19, TNFα is involved in acute inflammatory reactions and acts as an inflammation amplifier [18]. It also mediates lung inflammation and acute respiratory distress syndrome by reducing CD4 + and CD8 + T-cell levels, which are essential to the host’s defense against respiratory viruses in patients with severe COVID-19 [19–21]. Hussel et al. reported that TNFα depletion reduced the pulmonary recruitment of inflammatory cells, cytokine production by T cells, and the severity of illness without preventing virus clearance [22].

Several studies on antibody titers against the SARS-CoV-2 vaccine for patients with IBD have demonstrated that antibody titers were lower in patients treated with anti-TNFα antibody than in those without it [23, 24]. On the other hand, it has been reported that the T-cell immune response, even in patients with IBD receiving immunomodulators and biologics, was not different from that of healthy individuals [23, 24]. Taken together, these data strongly support the clinical course that anti-TNFα agents do not worsen the COVID-19 infection. Alhalabi et al. reported a case of a UC patient with COVID-19 pneumonia who was safely treated with an anti-TNFα agent [25]. However, data remain limited on the efficacy and the safety of anti-TNFα treatment in patients with COVID-19, based on large-scale clinical trials.

There is no clear consensus as to whether IBD patients with COVID-19 should continue IBD treatment. A sub-analysis of J-COSMOS data showed that neither continuation nor discontinuation of IBD medications affected COVID-19 severity, and that discontinuation of medications did not contribute to UC flares during COVID-19 [26]. However, considering its potential contributions of controlling intestinal inflammation in IBD patients to prevent SARS-CoV-2 infection and controlling COVID-19 exacerbations, aggressive therapeutic intervention is warranted in patients with highly active IBD, and patients with refractory IBD should continue the current treatment even if they develop COVID-19.

In this case, we first treated the patient with IFX-BS alone and finally required a combination therapy of IFX-BS and AZA to induce remission. SECURE-IBD and J-COSMOS data showed that IBD patients receiving corticosteroids and a combination of anti-TNFα antibody and immunosuppressive drugs at the time of diagnosing COVID-19 pneumonia had a higher severity rate of COVID-19 compared to patients receiving anti-TNFα antibody monotherapy [5, 8]. Previous reports have also shown that AZA attenuates the immune response to the virus [27, 28]. Therefore, we initially used IFX-BS alone. However, her symptoms deteriorated after the second infusion of IFX-BS because of the therapeutic impact of the antibody production against IFX-BS, owing this to its characteristics as chimeric antibody [29]. We added AZA to prevent loss of response after confirming the negativity of SARS-CoV-2 by PCR. Consequently, her intestinal symptoms improved without worsening the COVID-19 pneumonia.

In summary, we encountered a case of an older patient with moderately active UC and COVID-19 pneumonia who was successfully treated with an anti-TNFα agent and AZA. The current case suggests the importance of immunomodulatory therapy in patients with active UC while appropriately monitoring the COVID-19 infection. However, we need to accumulate cases similar to our case to evaluate the effectiveness and safety of the combination therapy of anti-TNFα agents and immunosuppressive drugs for patients with IBD who have COVID-19.

## Data Availability

The data underlying this article will be shared on reasonable request to the corresponding author.

## Abbreviations

ANCA: Anti-neutrophil cytoplasmic antibody
5-ASA: 5-Aminosalicylic acid
AZA: Azathioprine
COVID-19: Coronavirus disease 2019
IBD: Inflammatory bowel disease
IL: Interleukin
IFX-BS: Infliximab biosimilar
J-COSMOS: Japan COVID-19 surveillance in inflammatory bowel diseases
NLRP3: NLR family pyrin domain containing 3
SARS-CoV-2: Severe acute respiratory syndrome coronavirus 2
SECURE-IBD: Surveillance Epidemiology of Coronavirus Under Research Exclusion for Inflammatory Bowel Disease
SpO2: Peripheral capillary oxygen saturation
UC: Ulcerative colitis
TNFα: Tumor necrosis factor alpha
